# Stewart-Treves Syndrome Involving Chronic Lymphedema after Mastectomy of Breast Cancer

**DOI:** 10.1155/2017/4056459

**Published:** 2017-02-09

**Authors:** Smain Nabil Mesli, Amin Khayreddine Ghouali, Fouad Benamara, Fouzi Ahmed Taleb, Hicham Tahraoui, Chakib Abi-Ayad

**Affiliations:** ^1^Department of General Surgery “A”, Dr. Tidjani Damerdji University Hospital of Tlemcen, Tlemcen, Algeria; ^2^Experimental Surgery Laboratory N°38, Medical School of Tlemcen, University of Abou Bekr Belkaid, Tlemcen, Algeria

## Abstract

Steward-Treves syndrome is a cutaneous angiosarcoma that usually appears after long evolution of a lymphoedema after mastectomy for mammary neoplasia associated with an axillary dissection. This is a rare disease develop most of the time in upper arm and often confounded with cutaneous metastasis. Only the biopsy and immunohistochemical study confirm the diagnosis. The treatment is surgical and consists of large cutaneous excision, an amputation of the limb or even its disarticulation and will be followed by chemotherapy. Despite the treatment, the prognosis remains severe with poor survival. We report the case of a patient who had a Steward-Treves syndrome 20 years after lymphoedema following a left mastectomy with axillary dissection.

## 1. Introduction

In 1948, Stewart and Treves reported for the first time a series of six cases of cutaneous angiosarcoma developed in lymphedema of the upper limb after mastectomy with axillary dissection for breast cancer. The disease appears eight to twenty-four years after the occurrence of lymphedema [1]. These tumors are rare, less than 1% of all sarcomas [2, 3], and have high clinical aggressiveness and poor prognosis.

Up to date, more than 400 cases of Steward-Treves syndrome (STS) have been reported in the literature. The lymphatic stasis has diverse origins: congenital origin, postradiation origin, trauma, or burn [4]. The mechanism and pathophysiology of this neoplasia remain unknown at present.

## 2. Case Presentation

A 41-year-old active woman with no particular history presented with left breast carcinoma tumor in 1987. Biological and radiological clinical assessments had revealed no distant extension.

The patient underwent a surgical treatment of Patey technique in the left breast with axillary dissection. The anatomopathological study revealed that it was an invasive galactophoric adenocarcinoma and five to 23 positive nodes were detected. Four cycles of adjuvant chemotherapy with cyclophosphamide, doxorubicin, and 5-fluouracil were administered.

Seven years following the chemotherapy, a nodule appeared on the intervention scar. The biopsy confirmed a local recurrence of breast cancer. After a staging that was featureless, radiotherapy targeting the chest wall, the collarbone, and armpit with a dose of 45 Gy for five weeks was performed. Hormone therapy with tamoxifen 20 mg per day was administered.

Two years after the treatment, the patient developed a significant lymphedema of the left arm, starting from the wrist and reaching the left shoulder, which required regular sessions of lymphatic drainage. 20 years after the appearance of the left mammary neoplasia and the occurrence of chronic lymphedema of the left arm, a burn of second degree appeared on the affected limb. A local treatment was administrated.

**Figure 1:**
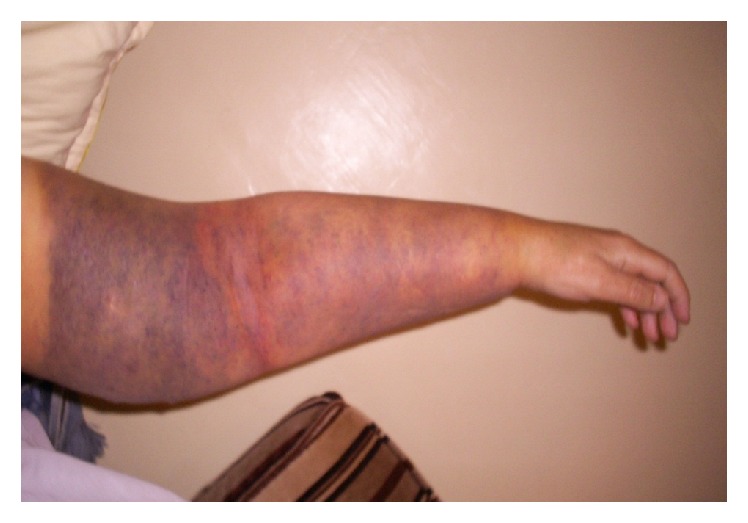
Left arm: mauve color with transitional tracer.

**Figure 2:**
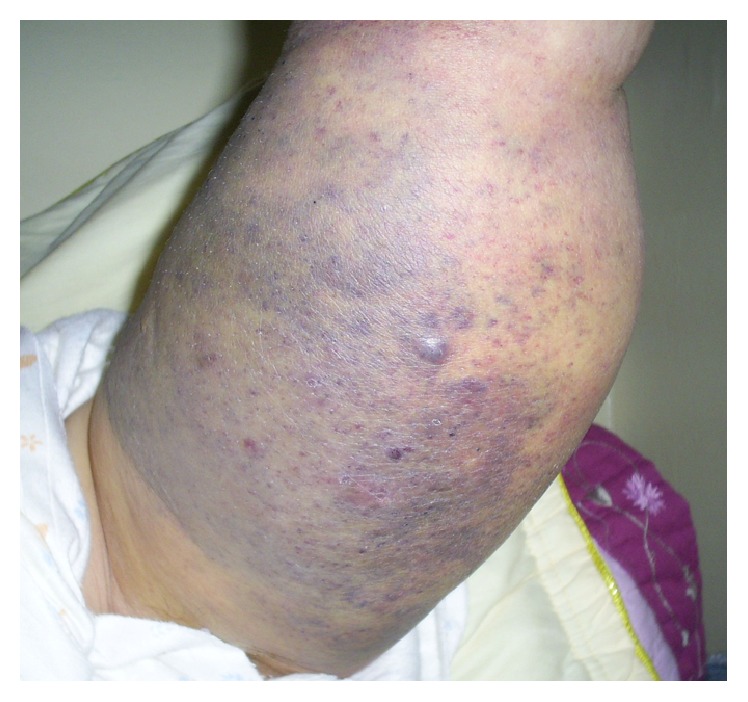
Appearance of the indurated nodule at the posterior surface of the upper limb of the left arm.

In September 2007 (one month after healing), the patient began to complain about a small hard erythematous-violet nodule at the rear and upper side of the left arm and reaching the posterior brachial region. They increased and became more painful and had a hard-bloody consistency on contact causing total functional left upper limb impotence (Figures 1 and 2).

**Figure 3:**
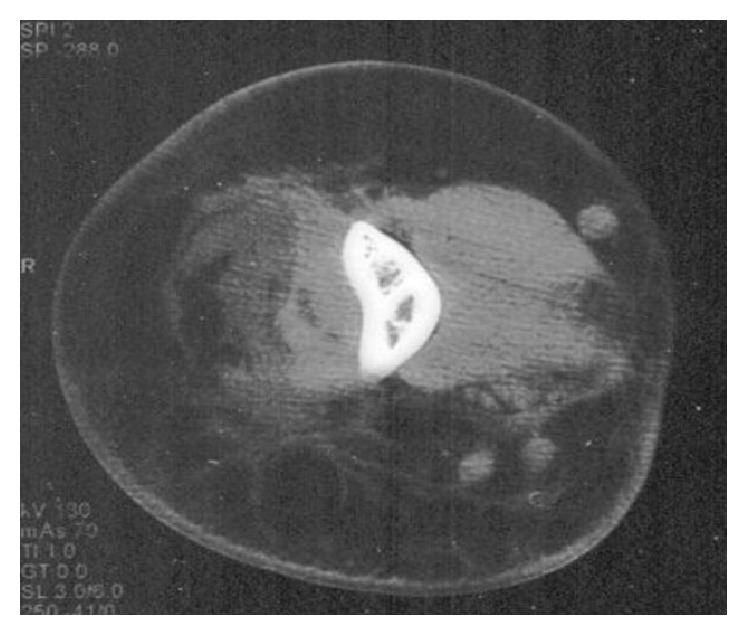
CT scan of left arm: fluidic collection with heterogeneous rearrangement of the fatty tissue with septa thickened and presence of muscular necrosis.

**Figure 4:**
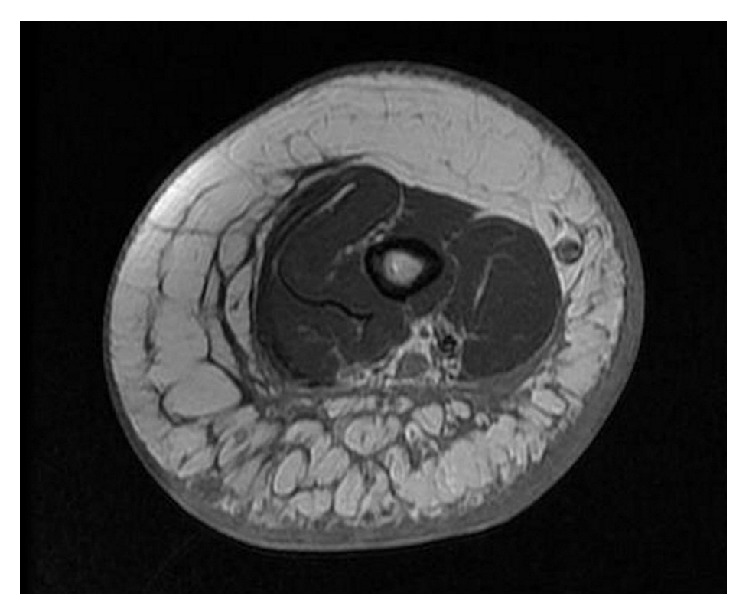
MRI of the soft parts of the upper left limb objectivating edematous infiltration of the fatty tissue with the presence of nodular formation opposite the brachial artery.

The patient was hospitalized for additional tests following a radiography of the left arm showing significant swelling of the soft tissues. An exam of the biological tumor markers (CA 19-9) indicated that those were in range. An ultrasound of the soft tissue showed a significant subcutaneous edema. A computed tomography scan revealed a member with a fluid collection under fascial muscular near the lower third of the humerus and diffused a thick septa anterosuperior arm (Figure 3), while an upper limb MRI showed the presence of a nodular formation in fatty tissue of the lower third of the arm (Figure 4).

A biopsy of the soft parts of the upper limb and brachial region was performed and the histological analyses confirmed the diagnosis of cutaneous angiosarcoma of chronic lymphedema. Immunohistochemistry indicated the vascular nature of the tumor thus eliminating the diagnosis of cutaneous metastases. Following this diagnosis, a complete staging was performed. The abdominal ultrasound and thoracoabdominal and bone scans revealed no remote extension. A radical decision was made including a dislocation of the left shoulder followed by two cycles of chemotherapy. The evaluation marked alteration of the general condition and dyspnea related to tumor spread in the lungs (pleurisy abundance). The histological tests revealed metastatic character of angiosarcoma. In April 2008, approximately eight months after the start of symptoms, the patient developed multiorgan failure that resulted in her death.

## 3. Discussion

The angiosarcoma is a rare and aggressive malignant tumor which develops in the vascular tissue and affects about 0.07% of breast cancer patients who survive five years at least after a mastectomy for Fitzpatrick [5] and 0.03% of patients surviving 10 or more years after radical mastectomy for Taghian et al. [6].

The main risk factor that could result in the development of an angiosarcoma is the lack of lymphatic drainage after axillary dissection due to radiation therapy [6]. Schreiber et al. [7] confirmed the role of chronic edema in vascular carcinogenesis. Other cofactors could be the mutation of a tumor suppressor gene p53 [2, 8]. The lymphatic stasis could cause anomaly traffic immunocompetent cells in the affected region as well as immune dysfunction in chronic lymphedema. The period between the onset of lymphedema and the STS's lesions was reported to vary between five and 11 years [9]. In our case the latency period was 13 years, which corresponds to identified publications.

In 90% of the cases of STS, lesions occur on the upper left limb [10]. Lesions appear in descending order: in the arm, forearm, wrist, and anterior thorax. This corresponded to our case. Many series reported that the lesions had bluish hematoma aspect and were followed by the appearance of red or blue nodules surrounded by an ecchymosis halo.

Chopra et al. [11] have diagnosed the tumor by magnetic resonance and have reported the usefulness of the MRI for evaluation of lesion's depth and orientation for biopsies. If there is a clinical suspicion of STS, a surgical biopsy is needed to confirm the angiosarcoma diagnosis and rule out others such as breast cancer and skin metastases Kaposi's sarcoma. If the standard histological examination is sufficient, it can be completed by immunohistochemistry in order to confirm the epithelial nature of the tumor cells. In our observation, the immunohistochemistry examination of the tumor confirmed the cutaneous angiomatous nature of the lesion by the use of specific markers (CD31, CD34, and AC Antifactor VIII), thus ruling out cutaneous metastases diagnosis [12]. There is no standard treatment for STS but surgery and radiotherapy are often recommended. In terms of surgery, the wide excision aims at obtaining a free safety margin of any residual tumor as mentioned by Noguchi et al. [13]. Other older publications propose more radical surgery with amputation of the limb or even a dislocation of the shoulder, but that procedure is not consensual [12, 14]. No significant difference in survival between the two techniques was observed in a literature review on 166 cases of STS [15]. In our case, the dislocation of the left shoulder was performed rather than the amputation. Radiation therapy is an option as it has demonstrated efficacy, while other authors [16, 17] propose it in a neoadjuvant process. The use of chemotherapy with 5-fluorouracil, methotrexate, bleomycin, and/or a combination of actinomycin D, vincristine, doxorubicin, and cyclophosphamide has shown beneficial effects [18, 19]. Our patient received an adjuvant chemotherapy with only two drugs (5-fluorouracil, methotrexate).

The aggressive nature of the STS and the high risk of recurrence inevitably result in extension to the limb, the chest wall, and pleura and this has been observed in our patient.

Considering the diagnosis delay of these tumors, the high rate of local recurrence, and the rapid onset of metastases, survival is poor with a median of 19 to 30 months after diagnosis [20–22]. The five-year survival is almost 10% in spite of the various treatment modalities [21]. Our patient died after eight months of the beginning of symptoms despite a radical surgery follow-up by a chemotherapy.

## 4. Conclusion

Stewart-Treves angiosarcoma is a rare and aggressive tumor with poor prognosis; therefore, it is necessary to stress the importance of regular clinical monitoring for patients affected with chronic lymphedema through preventive measures and biopsies of suspicious lesions in order to have an early diagnosis and good therapeutic management for improved survival.

## References

[1] Stewart F. W., Treves N. (1948). Lymphangiosarcoma in postmastectomy lymphedema. A report of six cases in elephantiasis chirurgica. *Cancer*.

[2] Cozen W., Bernstein L., Wang F., Press M. F., Mack T. M. (1999). The risk of angiosarcoma following primary breast cancer. *British Journal of Cancer*.

[3] Weiss S. W., Goldblum J. R. (2008). *Malignant Vascular Tumors: Enzinger et Weiss*.

[4] Wendt T., Kietzmann H., Schubert C., Kaiserling E. (1988). Progressive lymphangiokeratoma and angiosarcoma (Stewart-Treves syndrome) in congenital lymphedema. *Der Hautarzt; Zeitschrift fur Dermatologie, Venerologie, und Verwandte Gebiete*.

[5] Fitzpatrick P. J. (1969). Lymphangiosarcoma and breast cancer. *Canadian Journal of Surgery*.

[6] Taghian A., de Vathaire F., Terrier P. (1991). Long-term risk of sarcoma following radiation treatment for breast cancer. *International Journal of Radiation Oncology, Biology, Physics*.

[7] Schreiber H., Barry F. M., Russell W. C., Macon W. L., Ponsky J. L., Pories W. J. (1979). Stewart-treves syndrome: a lethal complication of postmastectomy lymphedema and regional immune deficiency. *Archives of Surgery*.

[8] Malkin D., Li F. P., Strong L. C. (1990). Germ line p53 mutations in a familial syndrome of breast cancer, sarcomas, and other neoplasms. *Science*.

[9] Kirova Y. M., Feuilhade F., Calitchi E., Otmezguine Y., Le Bourgeois J. P. (1999). Stewart-Treves syndrome after treatment for breast cancer. *Breast*.

[10] Schindera S. T., Streit M., Kaelin U., Stauffer E., Steinbach L., Anderson S. E. (2005). Stewart-Treves syndrome: MR imaging of a postmastectomy upper-limb chronic lymphedema with angiosarcoma. *Skeletal Radiology*.

[11] Chopra S., Ors F., Bergin D. (2007). MRI of angiosarcoma associated with chronic lymphoedema: Stewart Treves syndrome. *The British Journal of Radiology*.

[12] Tomita K., Yokogawa A., Oda Y., Terahata S. (1988). Lymphangiosarcoma in postmastectomy lymphedema (Stewart-Treves syndrome): ultrastructural and immunohistologic characteristics. *Journal of Surgical Oncology*.

[13] Noguchi M., Hasegawa H., Tajiri K. (1987). Stewart-treves syndrome. A report of two cases with a review of Japanese literature. *The Japanese Journal of Surgery*.

[14] Clark M. A., Thomas J. M. (2003). Major amputation for soft-tissue sarcoma. *British Journal of Surgery*.

[15] Grobmyer S. R., Daly J. M., Glotzbach R. E., Grobmyer A. J. (2000). Role of surgery in the management of postmastectomy extremity angio sarcoma (Stewart-Treves syndrome). *Journal of Surgical Oncology*.

[16] DiSimone R. N., el-Mahdi A. M., Hazra T., Lott S. (1970). The response to Stewart-Treves syndrome to radiotherapy. *Radiology*.

[17] Kaufmann T., Chu F., Kaufman R. (1991). Post-mastectomy lymphangiosarcoma (Stewart-Treves syndrome): report of two long-term survivals. *British Journal of Radiology*.

[18] Berebichez-Fridman R., Deutsch Y. E., Joyal T. M. (2016). Stewart-treves syndrome: a case report and review of the literature. *Case Reports in Oncology*.

[19] Young R. J., Brown N. J., Reed M. W., Hughes D., Woll P. J. (2010). Angiosarcoma. *The Lancet Oncology*.

[20] Sharma A., Schwartz R. A. (2012). Stewart-Treves syndrome: pathogenesis and management. *Journal of the American Academy of Dermatology*.

[21] Woodward A. H., Ivins J. C., Soule E. H. (1972). Lymphangiosarcoma arising in chronic lymphedematous extremities. *Cancer*.

[22] Wierzbicka-Hainaut E., Guillet G. (2010). Stewart-Treves syndrome (angiosarcoma on lyphoedema): a rare complication of lymphoedema. *Presse Medicale*.

